# *SPDEF* enhances cancer stem cell-like properties and tumorigenesis through directly promoting *GALNT7* transcription in luminal breast cancer

**DOI:** 10.1038/s41419-023-06098-z

**Published:** 2023-08-26

**Authors:** Jingyuan Li, Xue Wan, Dan Xie, Hui Yuan, Qin Pei, Yanan Luo, Yiyu Chen, Jiawen Xian, Ting Ye

**Affiliations:** 1 Department of Laboratory Medicine, the Affiliated Hospital of Southwest Medical University, Sichuan, 646000 P. R. China; 2 Department of Pathophysiology, Mudanjiang Medical University, Heilongjiang, 157011 P. R. China

**Keywords:** Breast cancer, Cell migration, Transcriptional regulatory elements, Diagnostic markers

## Abstract

**Background:**

Luminal breast cancer (BC) is the predominant subtype of breast cancer with a sustained risk of late recurrence and death. Understanding the molecular mechanisms for the oncogenesis of luminal BC would improve the prognosis for this large subset of patients. *SPDEF* was reported to be dysregulated in breast cancers. However, the biological functions and underlying molecular mechanism of *SPDEF* in luminal BC remains largely unknown. The aim of the present study was to elucidate the potential roles of *SPDEF* underlying subtype-specific functions in BC, especially in luminal subtypes.

**Methods:**

The expressions and clinicopathological characteristics of *SPDEF* in luminal BC patients were evaluated bioinformatically. In vitro and in vivo assays were performed to investigate the oncogenic function and stemness maintenance of *SPDEF* in luminal BC. Chromatin immunoprecipitation (ChIP) and dual luciferase reporter assays were conducted to determine the transcription regulation of *GALNT7* by *SPDEF*. *GALNT7* levels in serum from luminal BC patients were further detected by enzyme-linked immunosorbent assay (ELISA).

**Results:**

*SPDEF* is markedly upregulated in luminal BC and positively associated with tumor progression and poor prognosis. Furthermore, we confirmed that *SPDEF* enhanced the proliferation, migration, invasion and stemness of luminal BC cells in vitro as well the tumorigenicity in vivo. Mechanistically, we demonstrated the stimulative effect of *SPDEF* on the progression and stemness of luminal BC, which is mediated by its directly transcriptional target *GALNT7*. Clinically, we verified that the *GALNT7* can be used as a noninvasive diagnostic marker. Noteworthy, the combined detection of serum *GALNT7* and traditional tumor markers can enhance diagnostic accuracy thus is of vital importance in the early diagnosis of luminal BC.

**Conclusions:**

Our study reveals a novel mechanism by which *SPDEF* transcriptionally activates *GALNT7* via directly binding to its promoter to promote cell proliferation, motility and stemness, and led to luminal BC tumorigenesis and poor prognosis.

## Introduction

Breast cancer (BC) is the most common cancer worldwide and the leading cause of cancer-related death among women [1]. The high heterogeneous and diversity of BC brings difficulty to mechanisms research and hinders the development of molecularly targeted drugs, thereby enhancing the mortality of BC patients [2]. BC could be clustered into molecular subtypes, including luminal, human epidermal growth factor receptor 2-positive (HER2+) and triple-negative breast cancer (TNBC) tumors based on the expression of the estrogen receptor (ER), progesterone receptor (PR), and the overexpression of the HER2. Hereinto, the most common subtype is the luminal BC, which represents approximately two-thirds of all breast cancer [3]. Luminal BC is typically ER+ and/or PR+ and is generally associated with a better prognosis [4, 5]. While initial ER and/or PR-dependent treatment can be promising, the long-term efficacy of endocrine therapy is diminished by the recurrence of resistant tumors that have lost dependence on estrogen for growth [6]. Therefore, the survival of luminal BC patients is no better than that of patients with basal-like tumors after 20 years of follow-up [7]. Understanding the molecular mechanisms for the oncogenesis of luminal BC should lead to the identification of new molecules for targeted therapy that would improve the prognosis for this large subset of patients.

*SPDEF* (SAM pointed domain containing ETS transcription factor), belongs to the ETS (E26 transformation-specific) transcription factor family [8, 9], whose expression is largely restricted to epithelial tissues, including the lung, stomach, colon, and hormone-regulated epithelia such as the prostate, breast, and ovary [10–12]. *SPDEF* plays fundamental roles in mucus secretion, goblet cell differentiation, and tumorigenesis [11]. However, the roles of *SPDEF* in tumor progression currently remain controversial. The expression and molecular functions of *SPDEF* vary in different tumors. *SPDEF* is overexpressed and promotes the tumorigenesis and metastasis of ovarian and gastric cancers [11, 13], whereas it can inhibit prostate and colorectal tumor progression [14, 15]. In particular, the expression, biological role and the clinical significance of *SPDEF* in BC remains elusive. Several studies have demonstrated that *SPDEF* expression is lost in invasive BC cancer cell lines, supporting a tumor-suppressive function [16–18]. Conversely, *SPDEF* expression is up-regulated during BC progression, associated with increased tumor aggressiveness, suggesting instead a possible oncogenic role [19, 20]. Thus, it is great important to investigate the potential mechanisms of *SPDEF* underlying subtype-specific functions in BC.

In this study, we verified the oncogenic role of *SPDEF* in luminal BC progression and its clinicopathologic and prognostic importance in patients with luminal BC. Moreover, we revealed a novel mechanism by which *SPDEF* transcriptionally activates *GALNT7* via directly binding to its promoter to facilitate proliferation, motility and stemness of luminal BC cells in vitro as well as tumorigenicity in vivo. Finally, we found that *GALNT7* exerted a tumorigenic role which served as a promising no-invasion biomarker for combined diagnosis.

## Materials and Methods

### Gene expression profiling interactive analysis (GEPIA database)

GEPIA [21, 22] (http://gepia2.cancer-pku.cn/#survival) provides website analysis functions, including the gene expression analysis of tumor/normal, differential analysis of cancer types or pathological stages and survival analysis. The expression and relapse-free survival analysis (RFS) analysis of *GALNT7* in BC were analyzed by GEPIA.

### TCGA database analysis

mRNA expression in human BC subtypes was analyzed by TCGA Research Network (http://cancergenome.nih.gov). The original data from the TCGA database was normalized and analyzed by the edgeR analysis method [23]. To analyze the overall survival of patients with BC subtypes, patient samples were analyzed by Kaplan-Meier analysis.

### Immunohistochemistry (IHC)

Tissue specimens were collected from 128 participants, which involved 59 adjacent tissues and 69 luminal BC cases. Paraffin-embedded BC tissues were obtained from the Pathology Department of the Affiliated Hospital of Southwest Medical University. Firstly, paraffin-embedded tissue slides were dewaxed and blocked with normal goat serum (ZSGB-BIO, China) for nonspecific staining treatment at temperature for 30 min, after which they were treated with the primary antibody rabbit anti-SPDEF (1:200) (ABclone, USA) overnight at 4 °C. Subsequently, the slides were treated with biotinylated anti-rabbit IgG secondary antibody and next with horseradish peroxidase-conjugated streptavidin complex (ZSGB-BIO, China). Finally, the slides were incubated with adiaminobenzidine chromogen (DAB) solution and counterstained with hematoxylin to develop the colour reaction. Patient Consent Forms were obtained according to protocols approved by the Institutional Review Board of the Affiliated Hospital of Southwest Medical University. The H-score, or histochemical score was used to conduct quantitative assessment in immunohistochemistry (IHC). The staining intensity is scored on a scale of 0 to 3 (with 0 being no staining, 1+ being weak staining, 2+ being moderate staining, and 3+ being strong staining). The percentage of cells stained at each intensity level is then determined. H-score = (Percentage of cells stained at 1+ intensity) + (2 x Percentage of cells stained at 2+ intensity) + (3 x Percentage of cells stained at 3+ intensity).

### Survival analysis in Kaplan-Meier plotter database

To analyze the survival rates of patients with luminal BC, the prognostic significance of mRNA expression was evaluated using Kaplan-Meier Plotter [24, 25] (http://kmplot.com/analysis), which contained gene expression data and survival information of luminal BC patients.

### Cell culture

Normal human mammary cells MCF10A and human breast cancer cell lines, MCF7, T47D and BT-474 cell lines were purchased from the Cell Bank of the Chinese Academy of Sciences (Shanghai, China). MCF10A were cultured in special medium (Procell, China). MCF7 and T47D cells were maintained in DMEM high glucose medium (Hyclone, USA) supplemented with 10% fetal bovine serum (Gibco, USA). BT-474 cell were maintained in RPMI-1640 (Gibco, USA) supplemented with 20% fetal bovine serum (Gibco, USA), 10 μg/mL Insulin (Beyotime, China) and 2mM L-glutamine (Procell, China). Cells were incubated at 37 °C in an atmosphere of 5% CO2.

### RNA extraction and quantitative reverse transcription PCR (RT‐qPCR) analysis

Total RNA was extracted from cells using the Trizol (Takara, Japan) and chloroform. RNA concentration was measured using NanoDrop OneTM (Thermo Scientific, USA). And RNA converted into cDNA using PrimeScript RT reagent Kit (Takara, Japan). Quantitative PCR (qPCR) was conducted using the TB Green™ Premix Ex Taq™ II (Takara, Japan) in the Bio‐Rad CFX96 system according to the manufacturer’s protocol. The sequences of PCR primers are listed in Table S1.

### Stable knockdown of genes by lentivirus infection

Stable knockdown of *SPDEF* and over-expression of *GALNT7* in breast cancer cells was achieved with infection of sh-SPDEF (GeneBiogist, China) in the presence of 8 µg/ml polybrene (Genepharma, China) for 24 h. 48 h post the infection, the efficiency of infection was determined by RT-qPCR. Corresponding negative control (NC) cell lines were established by infection of viruses expressing empty vectors.

### Western blot

The proteins were extracted by Protein Extraction Kit (NCM Biotech, China). 50 µg protein sample was separated on 10% polyacrylamide gel (NCM Biotech, China) and transferred to PVDF membrane (Bio‐Rad, USA). Blocking the membrane in 5% notfat milk dissolved in TBST for 1-hour at room temperature. Then the membrane was hybridized in primary antibodies overnight at 4 °C on a shaker. The following primary antibodies were used: anti-SPDEF (1:7500 dilution, ABclone, USA), anti-GALNT7 (1:2500 dilution, TianQiShun, China) and anti-GAPDH (1:7500 dilution, Proteintech, USA). The membrane was incubated with the secondary antibodies diluted in TBST. Finally, the ECL system was used for HRP-conjugated secondary antibody (Bio-Rad, USA).

### Cell proliferation assay

Cellular proliferation was detected using a CCK-8 colorimetric assay kit (Beyotime, China). Luminal BC cells (2 × 10^3^/well) were seeded in 96-well plates to culture. CCK8 (10 μl) reagent was added to each well and the absorbance of each well at 450 nm was measured every 24 h with the multimode microplate reader (EnSpire, Singapore). Cell proliferation activity was continuously detected for 6 days.

### Wound scratch assay

The scratch test was used for cell migration assay. 3 × 10^5^ cells were seeded into a 6-well plate and cultured in medium with 10% FBS. When the cells grew to 80% confluence, the scratch was scored monolayer cells with sterile pipette tip, and continue to incubate with serum-free medium.

### Transwell migration and invasion assays

Transwell chambers were used to execute cell invasion assay with Matrigel (60 μl, 1:10 dilution in serum-free medium, Corning, USA) and migration assay without Matrigel. Liminal BC cells (5 × 10^4^/well) were seeded in the upper chamber in 100 μl of serum-free medium, at the same time, 600 μl medium with 10% FBS was added to the lower chamber as a chemoattractant. After incubation at 37 °C for 24 h, the upper surface of the membrane was gently removed with a swab. While the cells invaded the lower surface, the membrane was stained with 0.1% crystal violet. The stained cells in 3 randomly selected fields were counted.

### Transient overexpression assay

hGALNT7 pReceiver‐M98 and negative control were purchased from GeneCopoeia (GeneCopoeia, USA). Transient overexpression assay was performed and optimized using Lipo8000^TM^ (Beyotime, China) and MEM medium (Gibco, USA) according to the manufacturer’s protocol.

### Gene set enrichment analysis (GSEA)

GSEA software [26, 27] (http://www.gsea-msigdb.org/gsea/index.jsp) and C2 gene sets (curated gene sets) were used to analyze the TCGA Luminal BC dataset.

### Clinical characteristics and differential analysis of stemness indices

The stemness indices, mRNAsi (mRNA expression-based stemness index) and mDNAsi (DNA methylation-based stemness index) were uesd to describe the similar features between cancer cells and stem cells and it might be considered as the stemness indices of Cancer Stem Cells (CSCs) [28]. TCGA luminal BC samples were divided into two groups according to mRNA expressions and the mRNAsi and mDNAsi indices of the two groups were evaluated based on R analysis.

### Sphere formation assay

Luminal BC cells were plated at 1 × 10^3^ cells/well in low adhesion 6-well plates and were maintained in DMEM/F12 medium (Gibco, USA), supplemented with 2% B27 (Gibco, USA), 20 ng/ml Recombinant Human EGF (Peprotech, USA) and 20 ng/ml Recombinant Human bFGF (Peprotech, USA). After the cells were incubated for 12 days, the size and number of tumor spheres were calculated.

### Colony formation assay

MCF7 or T47D cells were digested with trypsin, suspended as a single cell and counted using the Bullboar counting plate. 1 × 10^3^ cells were seeded in 6-well plates and incubated for 1 week at 37 °C. Then, cells were washed with PBS, fixed with 4% Methanol for 30 min (mins) and stained for 10 min with 0.1% crystal violet.

### Subcutaneous tumor transplantation assay

Single-cell suspensions at designated concentrations were combined with equal volume of Matrigel Matrix (R&D Systems, USA). Then the mixture was injected subcutaneously into each side of the hind leg of BALB/c nude mice. After tumor cell transplantation, the tumor dimensions were measured every other day and the volume was calculated using V = (length × width^2)/2. When the volume reached about 1 cm [3], the mice were sacrificed and the neoplasm was excised for weighting. All protocols were approved by the Institutional Animal Care and Use Committee of Southwest Medical University and conducted with humane animal care.

### Flow cytometry

The expression of the ALDH1A1 marker was evaluated using flow cytometry, adhering strictly to the manufacturer’s protocol. We utilized the primary anti‐ALDH1A1 antibody at specific working dilutions (0.2 μg of 60171-1-Ig, Clone:1A10A2, sourced from Proteintech, USA). This was followed by the application of the secondary antibody, Fluorescein (FITC)–conjugated Affinipure Goat Anti-Mouse IgG(H + L), at a dilution ranging between 1:20 and 1:100 (also provided by Proteintech, USA).

### Drug sensitivity assays

CCK-8 colorimetric assay was used to detect the sensitivity of cells to paclitaxel in vitro. Cells were resuspended in a final concentration of 2 × 10^3^ cells/well, seeded into 96 well plates and subjected to paclitaxel after preincubation for 48 h. After 24 h of incubation withpaclitaxel, CCK-8 reagent (10 µl/well) was added and the plates were further incubated for 2 h. Subsequently, absorbance was determined at 450 nm by microplate reader.

### Chromatin immunoprecipitation (ChIP) assay

ChIP assay was performed to detect the molecular interactions of *SPDEF* with the promoter of *GALNT7* according to the manufacturer’s instruction (Beyotime, China). The primers of ChIP -qPCR listed in Table S2.

### Luciferase reporter

JASPAR software (http://jaspar.genereg.net/) was used to predict the presumptive binding sites of *SPDEF* that identified the *GALNT7* promoter region. The first three matching sequences were selected to verification. Luciferase reporter vectors containing the wild-type (WT) or mutant sequences (Mut) towards the *SPDEF* binding of the *GALNT7* promoter region were constructed (Tsingke, China). The pGL3-basic vector was included as the control. For luciferase activity assays, luminal BC cells were seeded in 24-well plates and cultured 24 h before transfection. Then cells were co-transfected with pGL3-basic or WT/Mut vector, and *SPDEF* over-expression plasmid/empty vector by EndoFectinTM-Max transfection reagent (GeneCopoeia, USA). The activity of Renilla plasmid was measured using the Dual-Luciferase Reporter Assay Kit (GeneCopoeia, USA).

### Enzyme-linked immunosorbent assay (ELISA)

Serum samples were collected from 64 participants, including 33 healthy control and 31 luminal BC patients without therapy, respectively. The study was carried out in accordance with the Institutional Review Board of the Affiliated Hospital of Southwest Medical University, and signed consent forms were obtained from each participant. *GALNT7* in human serum were detected by ELISA kit (Ruixin Biological Technology Co., Ltd, Quanzhou, China). *CEA* and *CA125* were quantified by Cobas e602 Electrochemiluminescence System (Roche, Switzerland).

### Statistical analysis

All data were analyzed by to the Shapiro-Wilk and Kolmogorov-Smirnov Test to check for normality, as well as the Levene’s test to verify the homogeneity of variances. Subsequent difference analyses were conducted by the t-test, chi-square test, Welch’s t-test, Wilcoxon or Mann-Whitney tests as appropriate using GraphPad Prism 7 and R, and presented as mean ± SEM. The *P* values less than 0.05 were considered statistically significant, *P < 0.05, **P < 0.01, ***P < 0.001. Correlation analysis was calculated with the Pearson correlation coefficient formula. Each experiment was replicated 3 times to ensure the reliability and reproducibility of the results.

## Results

### *SPDEF* is markedly upregulated in luminal BC and associated with tumor progression and poor prognosis

**Fig. 1:**
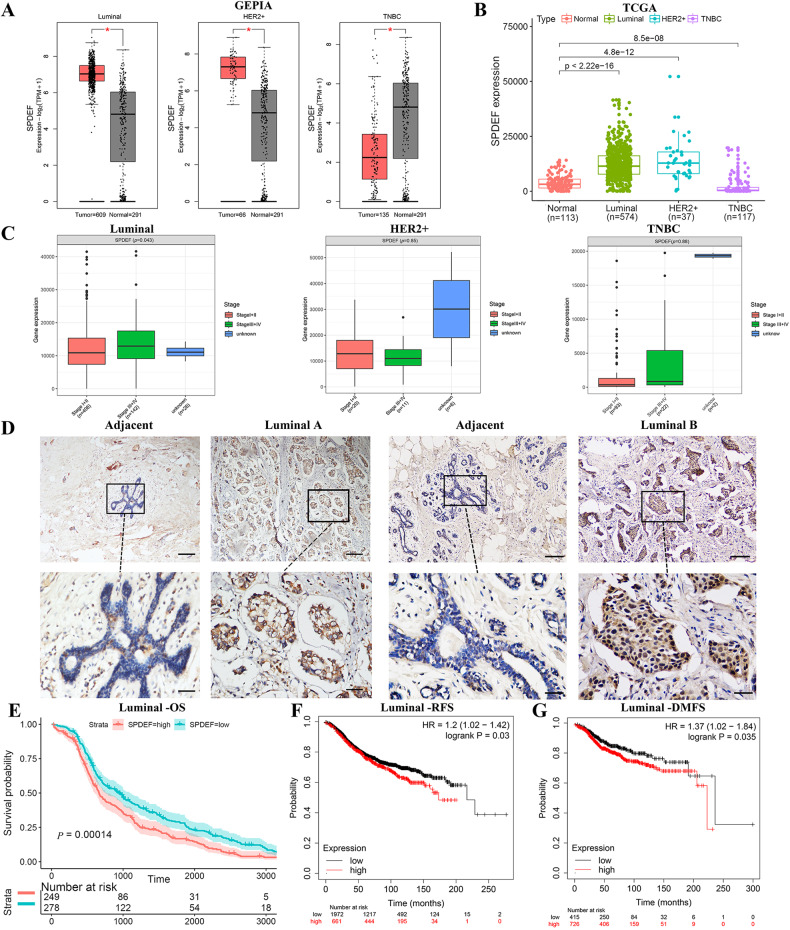
*SPDEF* is markedly upregulated in luminal BC and associated with tumor progression and poor prognosis. **A**–**B** *SPDEF* expression level in different subtypes of BC samples compared to noncancerous samples by GEPIA (**A**) and TCGA database (**B**). **C** Differential expressions of *SPDEF* in early, late and unknown tumor stage according to BC subtypes from TCGA database. **D** Representative immunohistochemical staining of *SPDEF* in luminal BC tissues, scale bar = 60 μm (upper panels), scale bar = 30 μm (lower panels). **E**–**G** Kaplan-Meier estimates of the OS (**E**), RFS (**F**), and DMFS (**G**) of patients with luminal BC according to *SPDEF* levels by TCGA and KM Plotter database.

Association between *SPDEF* and clinicopathological characteristics were performed to access the influence of *SPDEF* in breast cancer patients. We initially analyzed *SPDEF* mRNA expression in the publicly available datasets (GEPIA and TCGA). As showing in Fig. 1A, B, *SPDEF* mRNA expression was notably upregulated in luminal and HER2 + BC tissues, while significantly decreased in triple-negative BC (TNBC) compared to normal individuals. Importantly, *SPDEF* mRNA expression level was much higher in luminal BC patients with advanced stage (stage III ~ IV) disease compared with those with early stage (stage I ~ II) disease (Fig. 1C). However, *SPDEF* was not associated with tumor progression in the other two breast cancer subtypes (HER2 + BC and TNBC). Thus, we additionally compared the transcription levels of *SPDEF* among groups of luminal BC patients, according to different clinicopathological characteristics (Table 1). The results uncovered that there was no significant difference in the *SPDEF* expression level was observed in luminal BC cases with regard to different age, lymphoid nodal status and distant metastasis status. But remarkably, high *SPDEF* expression was positively associated with stage (*P* = 0.022) and tumor invasion (*P* = 0.031).

**Table 1:** The association of *SPDEF* mRNA expression and clinicopathological parameters in BC subtypes based on TCGA database.

Variables	*SPDEF* mRNA expression of luminal BC			*P* value
	Total (*n* = 574)	Low (*n* = 287)	High (*n* = 287)	
**Age at surgery**				
<51	159	77	82	0.641
≥51	415	210	205	
**cTNM Stage**				
I + II	406	214	192	**0.022**^a^
III + IV	142	59	83	
X	26	14	12	
**Tumor invasion**				
T1 + T2	472	246	226	**0.031**^a^
T3 + T4	95	38	57	
X	7	3	4	
**Lymphoid nodal status**				
-	245	133	112	0.101
+	317	150	167	
X	12	4	8	
**Distant metastasis status**				
M0	478	244	234	0.235
M1	10	7	3	
MX	86	36	50	

^a^Bold values indicate *P* < 0.05.

Further, to confirm the findings of bioinformatics analysis, we detected the protein expression of *SPDEF* in paraffin embedded tissues by immunohistochemistry (IHC) staining. As expected, luminal BC tissues were found to express higher levels of *SPDEF* protein than adjacent normal tissues and almost localized both in nucleus and cytoplasm (Fig. 1D and Table S3). Subsequently, to assess the prognostic potential of *SPDEF* in luminal BC, we found that high *SPDEF* expression was a significant indicator of poor overall survival (OS), relapse-free survival (RFS), and distance metastasis free survival (DMFS) (Fig. 1E-G). Taken together, these results suggest that the substantial increase of *SPDEF* expression contributes to the progression of luminal BC, leading to poor prognosis.

### *SPDEF* facilitates the proliferation, migration and invasion of luminal BC cells in vitro

**Fig. 2:**
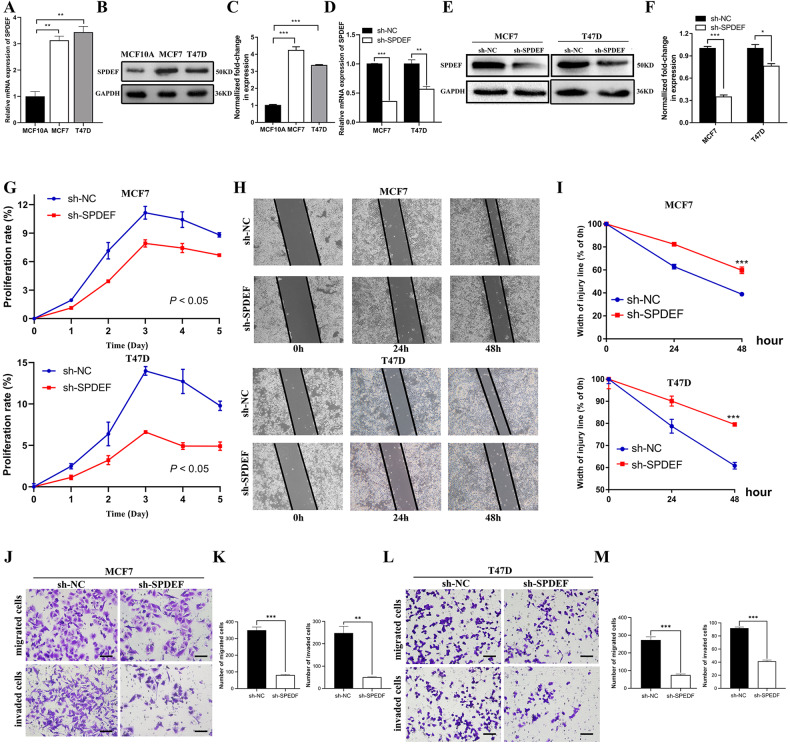
*SPDEF* facilitates the proliferation, migration and invasion of luminal BC cells in vitro. **A**–**C**. *SPDEF* abundance in MCF10A, MCF7 and T47D measured by RT-qPCR (**A**), protein abundance (**B**) and quantitative analysis (**C**) of *SPDEF* in MCF10A, MCF7 and T47D, the glyceraldehyde-3-phosphate dehydrogenase (GAPDH) was used as a reference control. **D** mRNA abundance of *SPDEF* in sh-NC and sh-SPDEF of MCF7 and T47D measured by RT-qPCR, sh-NC was the negative control, sh-SPDEF was the knockdown of SPDEF. **E**–**F** Protein abundance (**E**) and quantitative analysis (**F**) of *SPDEF* in sh-NC and sh-SPDEF measured by Western-blotting. **G** Proliferation activities of sh-NC and sh-SPDEF measured by the CCK-8 assay. **H**–**I**. Scratch wound healing assay results (**H**) and quantitative analysis (**I**). Images of all groups at 0 h, 24 h and 48 h time intervals post injury. J-M. Representative images of migrated and invaded cells and quantification assay of sh-NC and sh-SPDEF in MCF7 (**J**–**K**) and T47D cells (**L**–**M**), scale bar = 60 μm. **P < 0.01, ***P < 0.001.

To further characterize the biological functions of *SPDEF* in luminal BC cells, we firstly found that luminal BC cells, including MCF7, T47D and BT-474, had elevated *SPDEF* expression in comparison with MCF10A cells using RT-qPCR (Fig. 2A and Figure S1A) and western blot (Fig. 2B, C and Figure S1B, C). We then observed whether *SPDEF* knockdown could affect biological behaviors of luminal BC cells in which *SPDEF* expression was stably inhibited by RNA interference (sh-NC and sh-SPDEF groups). Effective inhibition of *SPDEF* expression was confirmed by RT-qPCR (Fig. 2D and Figure S1D) and western blot (Fig. 2E, F and Figure S1E, F), respectively. CCK-8 assay was used for detecting the cell viability of MCF7, T47D and BT-474 cells. Figure 2G and Figure S1G displayed that proliferation of luminal BC cells was significantly slowed down following transfection with sh-SPDEF. Subsequently, migration and invasion capacities were assessed via wound scratch and transwell assays. The data showed that the migration distances of MCF7 and T47D cells were decreased following transfection with shRNA targeting *SPDEF* (Fig. 2H, I). Furthermore, transwell assay demonstrated that *SPDEF* knockdown distinctly inhibited migration and invasion ability of luminal BC cells (Fig. 2J-M and Figure S1H-I). We further verified the remarkably increased mRNA (Figure S2A) and protein expression (Figure S2B, C) of *SPDEF* in MCF7 and BT-474 cells with *SPDEF* overexpression. Most importantly, overexpression of *SPDEF* significantly enhanced luminal cell proliferation (Figure S2D), invasive, and migratory (Figure S2E-J), confirming the oncogenic function of *SPDEF* in luminal BC.

### *SPDEF* promotes cancer stem cell-like features and tumorigenicity in luminal BC

**Fig. 3:**
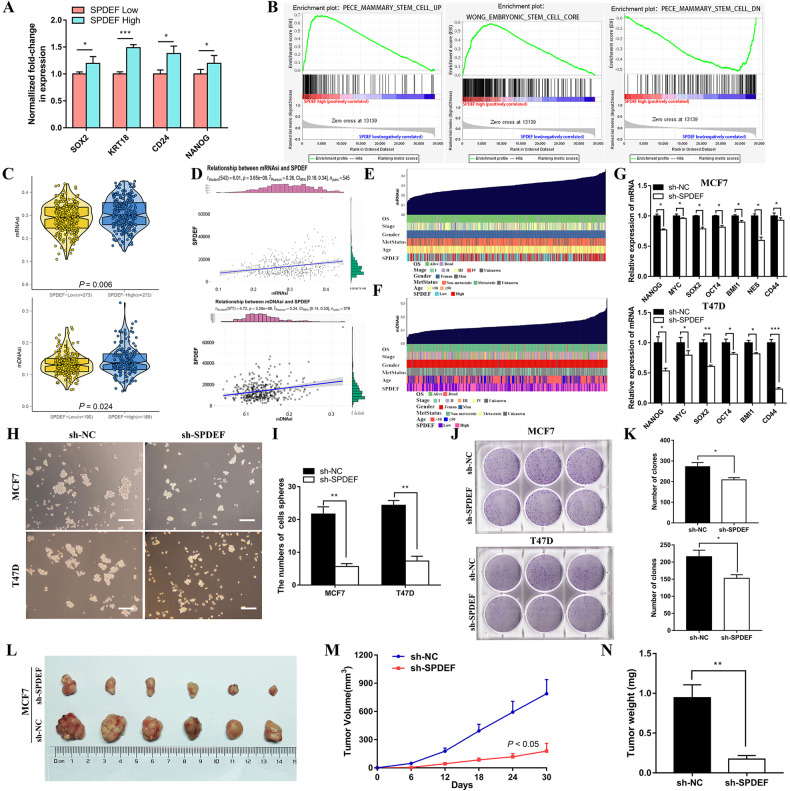
*SPDEF* promotes cancer stem cell-like features and tumorigenicity in luminal BC. **A** The differential expression of stem cell markers in high and low *SPDEF* expression groups. **B** GSEA assessment of the enrichment score profile of stemness gene set in the *SPDEF* high and low groups. **C** TCGA database analysis of mRNAsi and mDNAsi differences in *SPDEF* high and low groups. **D** Correlation of *SPDEF* expression and mRNAsi and mDNAsi in luminal BC. **E**–**F** An overview of the association between *SPDEF* and clinical features and mRNAsi (**E**) /mDNAsi (**F**) in luminal BC. **G** Expression of stem genes in sh-NC and sh-SPDEF of MCF7 and T47D cells by RT-qPCR. **H**–**I** The morphology of spheroid formation (**H**) and quantitative analysis (**I**) in sh-NC and sh-SPDEF of MCF7 and T47D cells, scale bar = 120 μm. **J**–**K** The soft agar colony formation assay (**J**) and quantitative analysis (**K**) in sh-NC and sh-SPDEF of MCF7 and T47D cells. **L** Tumor formation in nude mice following injection of control cells and genetically modified luminal BC cells (sh-NC and sh-SPDEF), respectively. **M**–**N** Tumor growth curves of luminal BC cells (sh-NC and sh-SPDEF) in nude mice. (**M**) Tumor volume. (**N**) Tumor weight. *P < 0.05, **P < 0.01, ***P < 0.001.

*SPDEF* was proved to act as a tumor-promoter in hepatocellular and lung cancers by maintaining the self-renewal of CSCs [29, 30]. Aimed to excavate the influence of *SPDEF* on stem cell-like properties, we firstly confirmed the expression of stem markers to verify the relationship between the expression level of *SPDEF* and stemness in luminal BC cases from TCGA database. The results revealed that the expression of embryonic stem cell markers (*SOX2*, *KRT18, CD24, NANOG*) were abundantly expressed in the high *SPDEF* group compared to that in the low group (Fig. 3A). Moreover, GSEA analysis showed that high *SPDEF* expression was positively associated with curated gene sets of stem cell in luminal BC samples from TCGA database (Fig. 3B). Further, we provide novel stemness indices for assessing the degree of oncogenic dedifferentiation in BC [28]. The stemness indices, mRNAsi and mDNAsi were remarkably elevated in high *SPDEF* expression group than that in low group (Fig. 3C). Notably, the overexpression of *SPDEF* was also positively correlated with above stemness indices (Fig. 3D). For mRNAsi and mDNAsi, by using the OCLR algorithm, the stemness index based on the gene expression profiles of 507(mRNAsi)/379(mRNAsi) luminal BC patients was calculated and then ranked from low to high to explore the associations between mRNAsi/mDNAsi and clinical features (Fig. 3E, F). As shown in Figure S3 patients in advanced stages had significantly higher mRNAsi and mDNAsi scores than those in early stage. Patients with poor clinical outcome demonstrated significantly higher mRNAsi scores, while patients aged ≤50 years had significantly higher mDNAsi scores than older patients. However, the stemness index scores both with mRNAsi and mDNAsi of metastatic status did not differ significantly among the groups.

To further elucidate the pivotal role of *SPDEF* in the stemness of luminal BC cells experimentally, the expressions of stemness genes were assessed between sh-NC and sh-SPDEF groups using RT-qPCR. The data indicated that *NANOG*, *MYC*, *SOX2*, *OCT4*, *BMI1*, *NES*, and *CD44* was remarkably reduced in sh-SPDEF cells compared to that in sh-NC cells (Fig. 3G). Next, we examined spheroid formation efficiency by the spheroid formation assay. *SPDEF* knockdown altered the size of spheroids and cell morphology (Fig. 3H), and greatly reduced the number of spheroids (Fig. 3I). Similarly, soft agar colony formation assay suggests that the number of clonogenic spheroids in the sh-SPDEF cells group was significantly lower than that in sh-NC cells group (Fig. 3J, K). In addition, the expression of ALDH1A1, the most widely used cell surface marker for isolating breast tumor-initiating cells, known as cancer stem cells, was also higher in MCF7 and BT-474 cells with over-expressed *SPDEF* compared to control group (Figure S4A, B). Drug sensitivity assays further revealed a higher paclitaxel (TAX) resistance in MCF7 and BT-474 cells with over-expressed *SPDEF* compared to control group. (Figure S4C, D). In vivo, 10^6^ MCF7-sh-NC and 10^6^ MCF7-sh-SPDEF cells were transplanted subcutaneously into the hinder leg of nude mice. Macroscopic observation of the subcutaneously injected nude mice revealed that the size of tumor nodes was smaller in the sh-SPDEF infected mice than in the sh-NC nude mice (Fig. 3L). Meanwhile, the growth rate of tumor volume (Fig. 3M) and the average weight of harvested nodules (Fig. 3N) were also inhibited in *SPDEF* knockdown tumors compared to control group. Notably, the stemness phenotype was further validated using an extreme gradient tumor formation assay in which MCF7 cells with up-regulated *SPDEF* were most efficient in tumor initiation compared with the control group, as evident by the sizes of the tumors and the growth kinetics of tumors (Figure S4E-G). Based on the above observations, we conclude that *SPDEF* promotes luminal cells self-renewal ability and clonogenic activity in vitro and in vivo.

### *SPDEF* was involved in cancer stem cell-like characteristics and tumorigenesis by regulating *GALNT7* expression in luminal BC

**Fig. 4:**
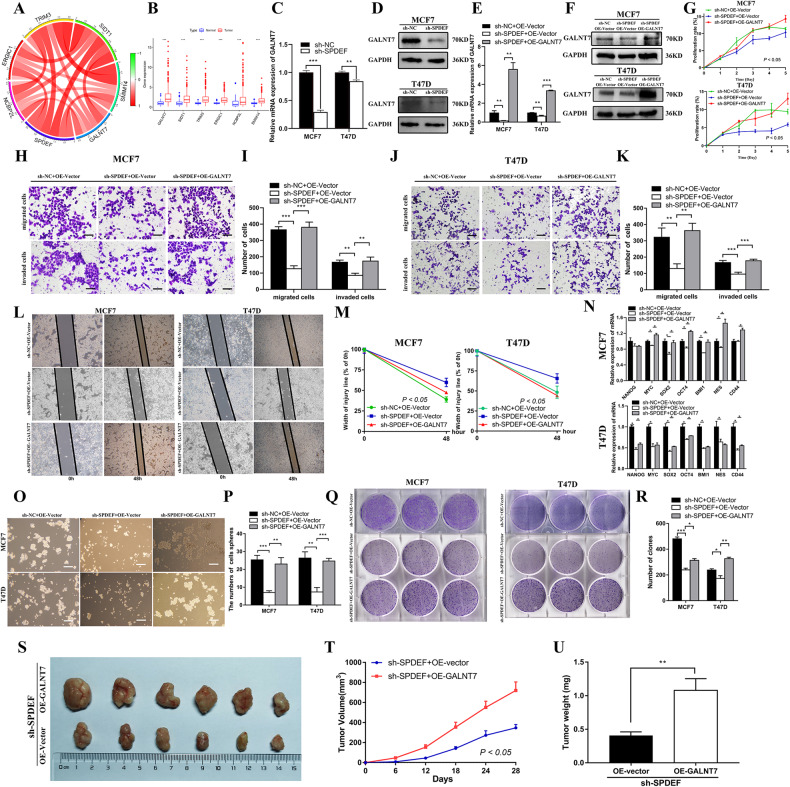
*SPDEF* was involved in cancer stem cell-like characteristics and tumorigenesis by directly regulating *GALNT7* expression in luminal BC. **A** Pearson test was performed to determine the correlation between *SPDEF* and six candidate genes in luminal BC. **B** The expression level of six candidate genes in luminal BC compared to non-cancerous samples by TCGA database. **C** *GALNT7* abundance in sh-NC and sh-SPDEF of MCF7 and T47D cells measured by RT-qPCR. **D** Protein abundance of *GALNT7* in sh-NC and sh-SPDEF measured by Western-blotting. **E** *GALNT7* abundance in shNC+OE-Vector, sh-SPDEF + OE-vector and sh-SPDEF + OE-GALNT7 cells measured by RT-qPCR. sh-NC was the negative control sh-RNA, sh-SPDEF was the knockdown of SPDEF, OE-vector was the negative control for the gene over-expression, OE-GALNT7 was the over-expression of GALNT7. **F** Protein abundance of *GALNT7* in shNC+OE-Vector, sh-SPDEF + OE-vector and sh-SPDEF + OE-GALNT7 cells measured by Western-blotting. **G** Proliferation activities of shNC+OE-Vector, sh-SPDEF + OE-vector and sh-SPDEF + OE-GALNT7 measured by the CCK-8 assay. H-K. Representative images of migrated and invaded cells and quantification assay of shNC+OE-Vector, sh-SPDEF + OE-vector and sh-SPDEF + OE-GALNT7 in MCF7 (**H**–**I**) and T47D cells (**J**–**K**), scale bar = 60 μm. **L**–**M** Scratch wound healing assay results (**L**) and quantitative analysis (**M**). Images of all groups at 0 h and 48 h time intervals post injury, scale bar = 120 μm. N. Expression of stem genes in shNC+OE-Vector, sh-SPDEF + OE-vector and sh-SPDEF + OE-GALNT7 of MCF7 and T47D cells by RT-qPCR. O-P. The morphology of spheroid formation (**O**) and quantitative analysis (**P**) in shNC+OE-Vector, sh-SPDEF + OE-vector and sh-SPDEF + OE-GALNT7 of MCF7 and T47D cells, scale bar = 120 μm. Q-R. The soft agar colony formation assay (**Q**) and quantitative analysis (**R**) in shNC+OE-Vector, sh-SPDEF + OE-vector and sh-SPDEF + OE-GALNT7 of MCF7 and T47D cells. **S**-**U**. Subcutaneous tumors were harvested after sh-SPDEF cells infected with OE-GALNT7 in BALB/c nude mice. **S** tumor volume, **T** tumor growth curve, **U** tumor weight. *P < 0.05, **P < 0.01, ***P < 0.001.

Since *SPDEF* is a transcription co-activator, it may promote liminal BC cells cancer stem cell-like properties and in vivo cancer biology characteristics by transcriptional activation of genes that are regulators of CSCs. Therefore, we further investigated to seek out possible targets of *SPDEF* that may regulate CSC-like properties in luminal BC cells. We identified six genes (*GALNT7*, *SIDT1*, *TRIM3*, *ERGIC1*, *NCBP2L*, and *SMIM14*) closely related to *SPDEF* from TCGA database (Fig. 4A). The histogram demonstrated that the most significant different expression of mRNA was *GALNT7* in six genes (Fig. 4B). We next examined the expression of *GALNT7* after *SPDEF* knockdown by RT-qPCR and Western blot. Down-regulation of *GALNT7* expression upon *SPDEF* inhibited was observed in MCF7 and T47D cells (Fig. 4C, D). Thus, we hypothesized that *GALNT7* could be a bona fide transcriptional target of *SPDEF*. To confirm that *GALNT7* is indeed the downstream target that mediating the tumor-promoting and stemness maintenance property of *SPDEF*, the expression of *GALNT7* was restored in *SPDEF* stably inhibited sh-SPDEF cells by overexpressing of *GALNT7* (Fig. 4E, F). The restoration of *GALNT7* expression in luminal BC cells reversed the ability of proliferation, migration and invasion, measured by CCK-8 assay (Fig. 4G), transwell assay (Fig. 4H-K) and scratch assay (Fig. 4L, M) in vitro. Further, we found the expressions of embryonic stem cell markers were significantly recovered after overexpression of *GALNT7* in sh-SPDEF group in luminal BC (Fig. 4N). Meanwhile, the restoration of *GALNT7* expression reverted sh-SPDEF cells back to clonogenic activity and self‐renewal ability in via serial spheroid formation assay (Fig. 4O, P) and clonogenic assay (Fig. 4Q, R). Most importantly, overexpression of *GALNT7* in sh-SPDEF cells can significantly restored tumorigenicity, confirming the regulation mechanism and its function in vivo (Fig. 4S-U). Conversely, down-regulation of *GALNT7* knock down after over-expressed *SPDEF* was observed in MCF7 and BT474 cells using RT-qPCR and Western-blotting (Figure S5A-C). In vitro, *GALNT7* inhibited reversed the enhanced proliferation (Figure S5D), migration (Figure S5E, F), invasion capabilities (Figure S5G-J) and even the cancer stem-like cell features due to *SPDEF* overexpression in luminal BC (Figure S6).

### *SPDEF* transcriptionally activates *GALNT7* via promoter binding

**Fig. 5:**
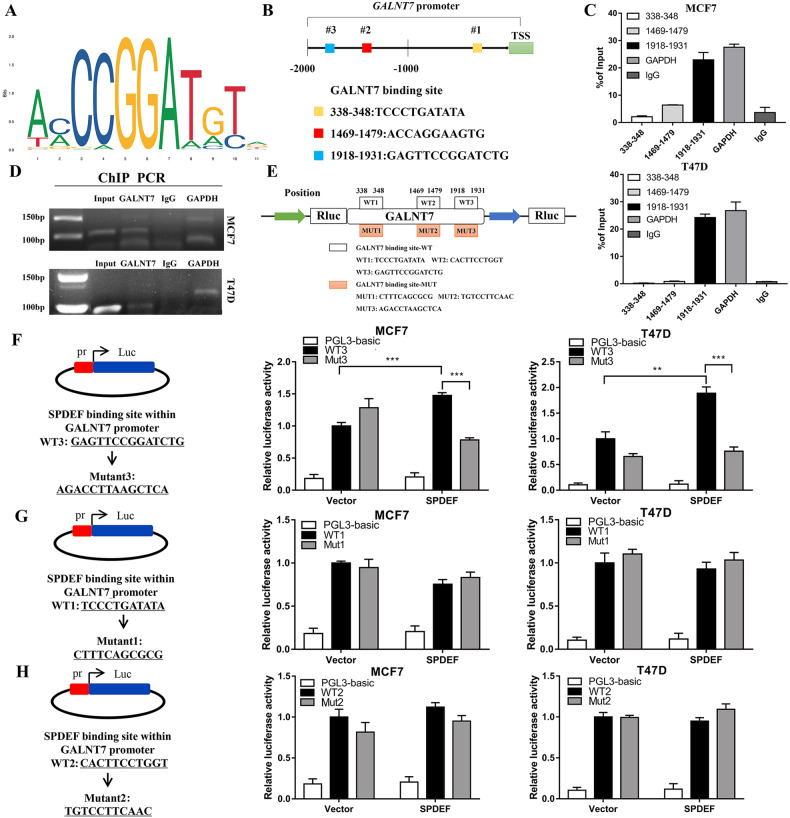
*SPDEF* transcriptionally activates *GALNT7* via promoter binding. **A** Motif of *SPDEF* binding sites. **B** Binding sites of *SPDEF* in the promoter region of *GALNT7*. **C** ChIP-qPCR analysis of the interaction of *SPDEF* with *GALNT7* promoter. **D** Agarose gel electrophoresis confirmed the binding site of *SPDEF* to the *GALNT7* promoter. *GAPDH* was used as a positive control. **E** Schematic diagram depicting the dual luciferase reporter assay for amplification of the regions. **F**–**H** *SPDEF* promoted *GALNT7* transcription through activating its promoter. (**F**) mutant 3 site. (**G**) mutant 1 site. **H** mutant 2 site. **P < 0.01, ***P < 0.001.

To address whether *SPDEF* could directly interact with the *GALNT7* prompter, we utilized the motif of *SPDEF* and promotor sequence of *GALNT7* to predict transcriptional factor binding site by bioinformatics in JASPAR database (Fig. 5A). We analyzed the region ~2200 bp upstream of the transcription start site (TSS) for *GALNT7* and identified three putative transcription factor-binding sites (Fig. 5B). Subsequently, we carried out chromatin immunoprecipitation (ChIP) assay to confirm the bioinformatics analysis. ChIP primers for the potential three sites were designed to amplify promoter regions containing putative binding sites, and the distal region primers were used as a negative control (Table S2). We conducted ChIP assay by incubating luminal BC cells nuclear extracts in the presence of anti-SPDEF, anti‐RNA Polymerase II antibody (as a positive control) or IgG (as a negative control). As shown in Fig. 5C and Figure S7A, *SPDEF* localized in +1918 ~ +1931 region of *GALNT7* promoter was responsible for the majority of its transcriptional activation activity compared with the IgG control in luminal BC cells by ChIP-qPCR. The DNA gel electrophoresis also exhibited the similar results (Fig. 5D and Figure S7B).

To further investigate whether *GALNT7* is transcriptionally induced by *SPDEF*, substitution mutations of the top three individual sites with the highest predicted scores were prepared for dual luciferase reporter assay (Fig. 5E). Abrogation of the mutant 3 site repressed luciferase activity in the luminal BC cells (Fig. 5F and Figure S7C). However, the two other mutant sites didn’t show significant decrease in transcriptional activity as compared with the vector control (Fig. 5G, H and Figure S7D, E). Taken together, these results indicated that *SPDEF* could directly bind to the predicted sites in the *GALNT7* promoter region +1918 ~ +1931 and were crucial for the transcriptional activation of *GALNT7* expression.

### *GALNT7* promotes stemness characteristics and is a potential diagnostic biomarker of luminal BC

**Fig. 6:**
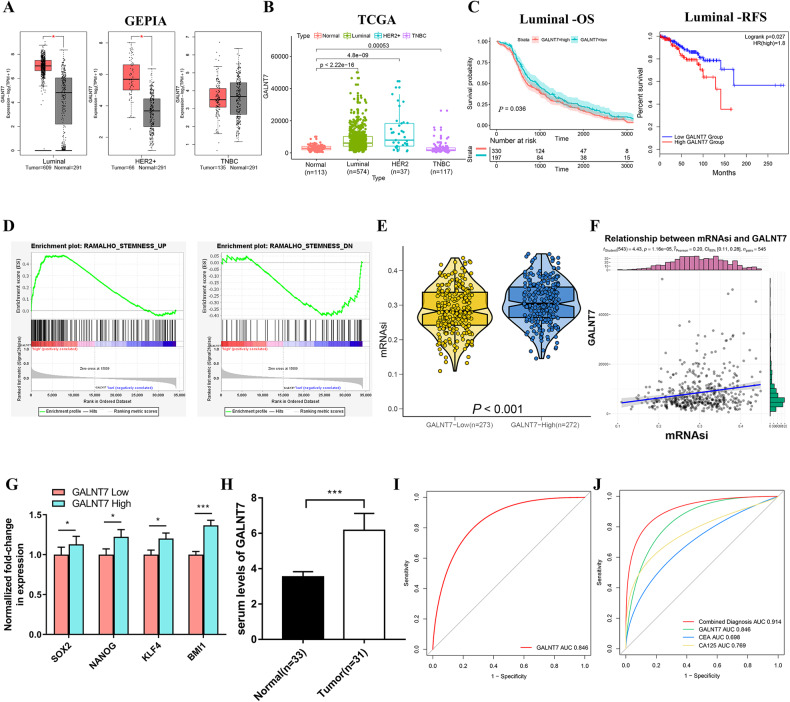
*GALNT7* is a potential diagnostic biomarker in luminal BC that modulates cancer stem cell properties. **A**, **B** *GALNT7* expression level in different subtypes of BC samples compared to non-cancerous samples by GEPIA (A) and TCGA database (**B**). **C** Kaplan-Meier estimates of the OS and RFS of patients with luminal BC according to *GALNT7* levels. **D** GSEA assessment of the enrichment score profile of stemness gene set in the *GALNT7* high and low groups. **E** TCGA database analysis of mRNAsi difference in *GALNT7* high and low groups. **F** Correlation of *GALNT7* expression and mRNAsi in luminal BC. **G** The differential expression of stem cell markers in high and low *GALNT7* expression groups. **H** Serum levels of *GALNT7* in luminal BC patients and healthy people. **I** ROC analysis for the *GALNT7* in luminal BC patient serum. J. ROC curves for diagnostic models of the combination of *GALNT7*, CEA and CA125. *P < 0.05, ***P < 0.001.

To determine the clinical relevance of *GALNT7* in BC, we analyzed the transcription levels of *GALNT7* by using GEPIA and TCGA database. As shown in Fig. 6A, B, *GALNT7* was remarkably overexpressed due to increased mRNA in luminal and HER2 + BC compared to normal individuals (Fig. 6A, B). Kaplan–Meier plots analysis revealed that luminal BC patients with high *GALNT7* expression levels had significantly worse overall survival and relapse-free survival in comparison with patients with low *GALNT7* expression level (Fig. 6C). Moreover, we investigated the relationship between elevated *GALNT7* and cancer stem cell-like properties in luminal BC. GSEA analysis showed that high *GALNT7* expression was positively associated with gene sets of stem cell (Fig. 6D). The mRNAsi was remarkably elevated in high *GALNT7* expression group than that in low group (Fig. 6E). Furthermore, *GALNT7* expression was positively correlated with mRNAsi scores in luminal BC patients (Fig. 6F), and the expression of stemeness markers (*SOX2*, *NANOG*, *KLF4*, *BMI1*) was significantly increased in the high *GALNT7* group compared to that in the low group (Fig. 6G). To further verify the clinical values of *GALNT7*, we examined the expressions in luminal BC serum by ELISA and quantized on a standard curve. *GALNT7* was upregulated in the serum of luminal BC patients compared with that in healthy individuals (Fig. 6H and Table S4). The area under the ROC curve (AUC) was 0.846, which also demonstrates that *GALNT7* has higher clinical value as a non-invasive biomarker in luminal BC (Fig. 6I). Meanwhile, the combined detection of *GALNT7*, *CEA* and *CA125* can improve the diagnosis efficiency of luminal BC with both better sensitivity (85.7%) and specificity (82.2%) (Fig. 6J). These results clearly indicate that *GALNT7* modulates cancer stem cell properties and confers well-diagnostic value in luminal BC.

## Discussion

Emerging evidence have demonstrated that the crucial role of *SPDEF* in the pathogenesis and progression of BC [31, 32]. However, the definition of *SPDEF* in the dichotomy of cancer-regulatory genes in BC has been controversial [33, 34]. Few studies focus on the mechanisms underlying the pro-oncogenic or tumor suppressive activity of *SPDEF* depending on different BC subtypes. Prior to this study, we have made a preliminary attempt to elucidate that *SPDEF* may play a diversity role in the expression levels, clinicopathologic importance, biological function and prognostic evaluation depending on different BC subtyping [35, 36]. However, the oncogenic function and downstream mechanisms of *SPDEF* in luminal BC are still unclear. Here, we reported the identification of *SPDEF* which represents a new mechanism of driving cancer stem cell-like properties and tumorigenicity of luminal BC that is mediated by *GALNT7*.

In the present study, the potent oncogene function of *SPDEF* in luminal BC has been elucidated by bioinformatics analysis and experimental evidence (Figs. 1, 2 and Figures S1–S3). Prior to this study, the cancer-promoting activity and clinical relevance of *SPDEF* in luminal BC has not been well explored and established. Only limited reports showed a correlation between *SPDEF* overexpression and malignant progression in luminal BC. In ER^+^ BC, *SPDEF* inversely regulated by ER and GATA3 is essential for tumorigenesis and is also required in models of endocrine-resistance [6]. In another report, high *SPDEF* expression enhances the tumorigenic growth of luminal BC cells and correlates with poor overall survival for BC patients with ER^+^ tumors [19]. Consistent with above observations, we revealed the highly expressed *SPDEF* was positively associated with the progression and poor prognosis in luminal BC. The first notable finding of our study is to provide convincing evidences supporting a rather definitive role of *SPDEF* in facilitating the proliferation, migration and invasion of luminal BC cells in vitro (Fig. 2 and Figures S1, S2) as well as promoting luminal BC oncogenesis and progression in vivo (Figs. 1 and 3L-N, Table 1). However, further in-depth mechanistic characterization and clinical research will answer whether *SPDEF* is a novel target for treatment and prognosis in luminal BC.

The second notable finding of this study is that we provided robust evidence for a regulatory role of *SPDEF* in the maintenance of luminal BC cancer stem cell-like properties. Specifically, we identified and molecularly concluded that *SPDEF* promotes luminal cells self-renewal ability and clonogenic activity in vitro (Fig. 3A-K and Figure S4A-D) and in vivo (Fig. 3L–N and Figure S4E-G). Accumulating evidence suggests that breast cancer progression might be driven by cancer stem cells that are more metastatic and refractory to conventional chemotherapeutics [37, 38]. Our knowledge of *SPDEF* on stemness properties comes mostly from studies involving the differentiation of goblet cell, Paneth cell, and normal mammary luminal cells [6, 39, 40]. A recent study reported that *SPDEF* could bind to the miR-448 promoter to downregulate DOT1L, whereby promoting self-renewal of hepatocellular carcinoma stem cells [30]. Nevertheless, the role of *SPDEF* in regulating cancer stem-like properties in luminal BC has not been established prior to the present study. This study demonstrated for the first time that *SPDEF* may promote cancer stem-like features and tumorigenicity in luminal BC, which will add new perspectives to the previously proposed oncogenic activity of *SPDEF*.

The third novelty is that we demonstrated the stimulative effect of *SPDEF* on progression and stemness of luminal BC, which is mediated by its direct transcriptional target *GALNT7* (Figs. 4, 5 and Figures S5, S6). *SPDEF* plays critical roles in many biological processes by directly activating downstream target gene transcription. To explore the potential molecular mechanism of action of *SPDEF* in luminal BC, bioinformatics analysis and experiments were conducted. We confirmed that *SPDEF* expression was positively correlated with *GALNT7* expression in luminal BC patients (Fig. 4A, B). Meanwhile, the direct targeting of *GALNT7* by *SPDEF* was confirmed by restoration of *GALNT7* in *SPDEF* silenced luminal BC cells which successfully reversed the cancer stem cell-like phenotype and oncogenic function in vitro and in vivo (Fig. 4C-U), and the other direction is also true (Figures S5, S6). Furthermore, we showed that *SPDEF* could directly bind to the *GALNT7* promotor to activate its expression by ChIP analysis and dual luciferase reporter assay (Fig. 5 and Figure S7). *GALNT7* has been reported as a member of the acetylgalactosaminyltransferase family which materializes a certain biological effect by regulating the interaction between tumor cells and the extracellular environment [41]. Few recent studies have shown the crucial role of *GALNT7* in tumorigenesis of colorectal cancer [42], cervical cancer [43], and prostate cancer [44, 45]. Prior to the present study, the role of *GALNT7* in regulating stemness and development of luminal BC was not elucidated. To our knowledge, this report is the first evidence that *SPDEF* regulated *GALNT7* to induce cancer cell traits in the luminal BC.

The fourth notable observation derived from this study is the high clinical relevance of *GALNT7* in luminal BC, which raises the potential of exploring the diagnosis and prognosis (Fig. 6). In our bioinformatics analysis, the difference expression and prognostic value of *GALNT7* was remarkably consistent with that of *SPDEF* in various BC subtyping (Fig. 6A–C). Furthermore, we provided evidence that *GALNT7* may play a similarly important role in regulating the cancer stem cell-like properties in luminal BC (Fig. 6D–G). Importantly, we found that the combined determination of serum *GALNT7* and traditional tumor markers (CEA and CA125) could enhance diagnostic accuracy thus is of vital importance in luminal BC (Fig. 6H–J). Therefore, the screening for biomarkers associated with cancer stem cell signatures provided novel insights into the selection of tumor diagnostic biomarkers.

Although we found that *SPDEF* might enhance cancer stem cell-like phenotype and tumorigenesis of luminal BC by activating *GALNT7* transcription, there are still some limitations to the current study. Firstly, IHC and serum sample analysis were conducted in a relatively small number of patients. A larger cohort should be used to further explore the relationship between *SPDEF* and/or *GALNT7* expressions and clinical features. Secondly, the mechanism by which *GALNT7* regulates stemness has not been explored. Further investigation is needed to elucidate this mechanism.

Taken together, this study revealed a novel role for *SPDEF* in luminal BC. We mainly demonstrated that *SPDEF* transcriptionally activates *GALNT7* via directly binding to its promoter to drive cancer stem cell-like properties and tumorigenicity in luminal BC. Our findings provide new insights into the mechanism of liminal BC tumorigenesis and a promising biomarker and therapeutic target for patients with liminal BC.

## Supplementary information

Supplementary Figure legend

supplement Figure S1

supplement Figure S2

supplement Figure S3

supplement Figure S4

supplement Figure S5

supplement Figure S6

supplement Figure S7

supplement TableS1

supplement TableS2

supplement Table S3

supplement Table S4

western blot

Reproducibility checklist

## Data Availability

The datasets presented in this study can be found in online repositories. The names of the repository/repositories and accession number(s) can be found in the article.
