# Supplementary material for: SPDEF enhances cancer stem cell-like properties and tumorigenesis through directly promoting GALNT7 transcription in luminal breast cancer
Source: Cell Death Dis. 2023 Aug 26;14(8):569. doi: 10.1038/s41419-023-06098-z (PMC10460425; doi:10.1038/s41419-023-06098-z)
Supplement: Supplementary file 1 — Supplementary Figure legend [file 41419_2023_6098_MOESM1_ESM.docx]

**Supplementary Figure legend**

**Figure S1**

A. *SPDEF* abundance in MCF10A and BT-474 measured by RT-qPCR. B. Protein abundance (B) and quantitative analysis (C) of *SPDEF* in MCF10A and BT-474. D. mRNA abundance of *SPDEF* and *GALNT7* in sh-NC and sh-SPDEF of BT-474 measured by RT-qPCR. E-F. Protein abundance (E) and quantitative analysis (F) of *SPDEF* and *GALNT7* in sh-NC and sh-SPDEF measured by Western-blotting. G. Proliferation activities of sh-NC and sh-SPDEF measured by the CCK-8 assay. H-I. Representative images of migrated and invaded cells (I) and quantification assay (I) of sh-NC and sh-SPDEF in BT474 cell lines, scale bar = 60μm. **P < 0.01, ***P < 0.001.

**Figure S2**

A. mRNA abundance of *SPDEF* in OE-Vector and OE-SPDEF of MCF7 and BT-474 measured by RT-qPCR, OE-vector was the negative control for the gene over-expression, OE-SPDEF was the over-expression of SPDEF. B-C. Protein abundance (B) and quantitative analysis (C) of *SPDEF* in OE-Vector and OE-SPDEF measured by Western-blotting. D. Proliferation activities of OE-Vector and OE-SPDEF of MCF7 and BT-474 measured by the CCK-8 assay. E-F. Scratch wound healing assay results (E) and quantitative analysis (F). Images of all groups at 0h, 24h and 48h time intervals post injury in of MCF7. G-J. Representative images of migrated and invaded cells and quantification assay of OE-Vector and OE-SPDEF in MCF7 (G-H) and BT-474 cells (I-J), scale bar = 60μm. ***P < 0.001.

**Figure S3**

A-D. Violin plot showed mRNAsi which was analysed by Age (A), Stage (B), OS Status (C) and Metastasis (D).

E-H. Violin plot showed mDNAsi which was analysed by Age (E), Stage (F), OS Status (G) and Metastasis (H).

**Figure S4**

A-B. The expression of CSCs marker ALDH1A1 in OE-Vector and OE-SPDEF of MCF7 (A) and BT-474 (B) used by flow cytometry. C-D. Drug sensitivity assays in OE-Vector and OE-SPDEF of MCF7 (C) and BT-474. IC50: Half Maximal Inhibitory Concentration. (D). E-G. Extreme gradient tumor formation assays in OE-Vector and OE-SPDEF of MCF7 in BALB/c nude mice. (E) tumor volume, (F) tumor growth curve, (G) tumor weight. *P < 0.05, ***P < 0.001.

**Figure S5**

A. *GALNT7* abundance in OE-Vector+shNC, OE-SPDEF+sh-NC and OE-SPDEF+sh-GALNT7 cells measured by RT-qPCR. OE-vector was the negative control for the gene over-expression, OE-SPDEF was the over-expression of SPDEF, sh-NC was the negative control sh-RNA, sh-GALNT7 was the knockdown of GALNT7. B-C. Protein abundance (B) and quantitative analysis (C) of *GALNT7* in OE-Vector+shNC, OE-SPDEF+sh-NC and OE-SPDEF+sh-GALNT7 cells measured by Western-blotting. D. Proliferation activities of OE-Vector+shNC, OE-SPDEF+sh-NC and OE-SPDEF+sh-GALNT7 measured by the CCK-8 assay. E-F. Scratch wound healing assay results (E) and quantitative analysis (F). Images of all groups at 0h, 24 and 48h time intervals post injury in MCF7. G-J. Representative images of migrated and invaded cells (G-H) and quantification assay (I-J) of OE-Vector+shNC, OE-SPDEF+sh-NC and OE-SPDEF+sh-GALNT7 in MCF7 and BT-474 cells, scale bar = 60μm. *P < 0.05, **P < 0.01, ***P < 0.001.

**Figure S6**

A-B. The expression of ALDH1A1 in OE-Vector+shNC, OE-SPDEF+sh-NC and OE-SPDEF+sh-GALNT7 of MCF7 (A) and BT-474 (B) used by flow cytometry. C-D. Drug sensitivity assays in OE-Vector+shNC, OE-SPDEF+sh-NC and OE-SPDEF+sh-GALNT7 of MCF7 (C) and BT-474 (D). IC50: Half Maximal Inhibitory Concentration.

**Figure S7**

A. ChIP-qPCR analysis of the interaction of *SPDEF* with *GALNT7* promoter in BT-474. B. Agarose gel electrophoresis confirmed the binding site of *SPDEF* to the *GALNT7* promoter in BT-474. C-E. *SPDEF* promoted *GALNT7* transcription through activating its promoter. (C) mutant 3 site. (D) mutant 1 site. (E) mutant 2 site. ***P < 0.001.
