# Supplementary figures and images for: SPDEF enhances cancer stem cell-like properties and tumorigenesis through directly promoting GALNT7 transcription in luminal breast cancer
Source: Cell Death Dis. 2023 Aug 26;14(8):569. doi: 10.1038/s41419-023-06098-z (PMC10460425; doi:10.1038/s41419-023-06098-z)

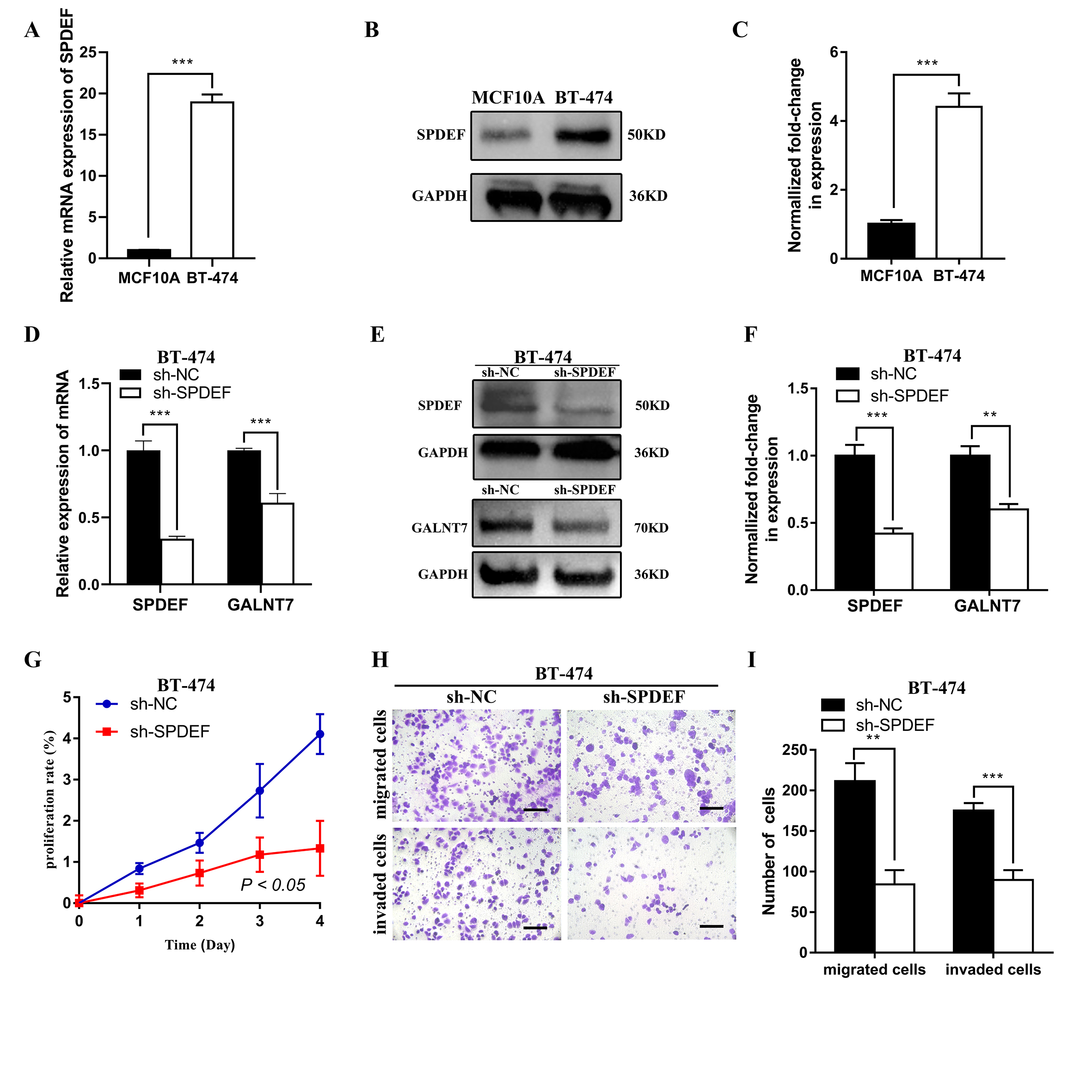

Supplement: Supplementary file 2 — supplement Figure S1 [file 41419_2023_6098_MOESM2_ESM.jpg]

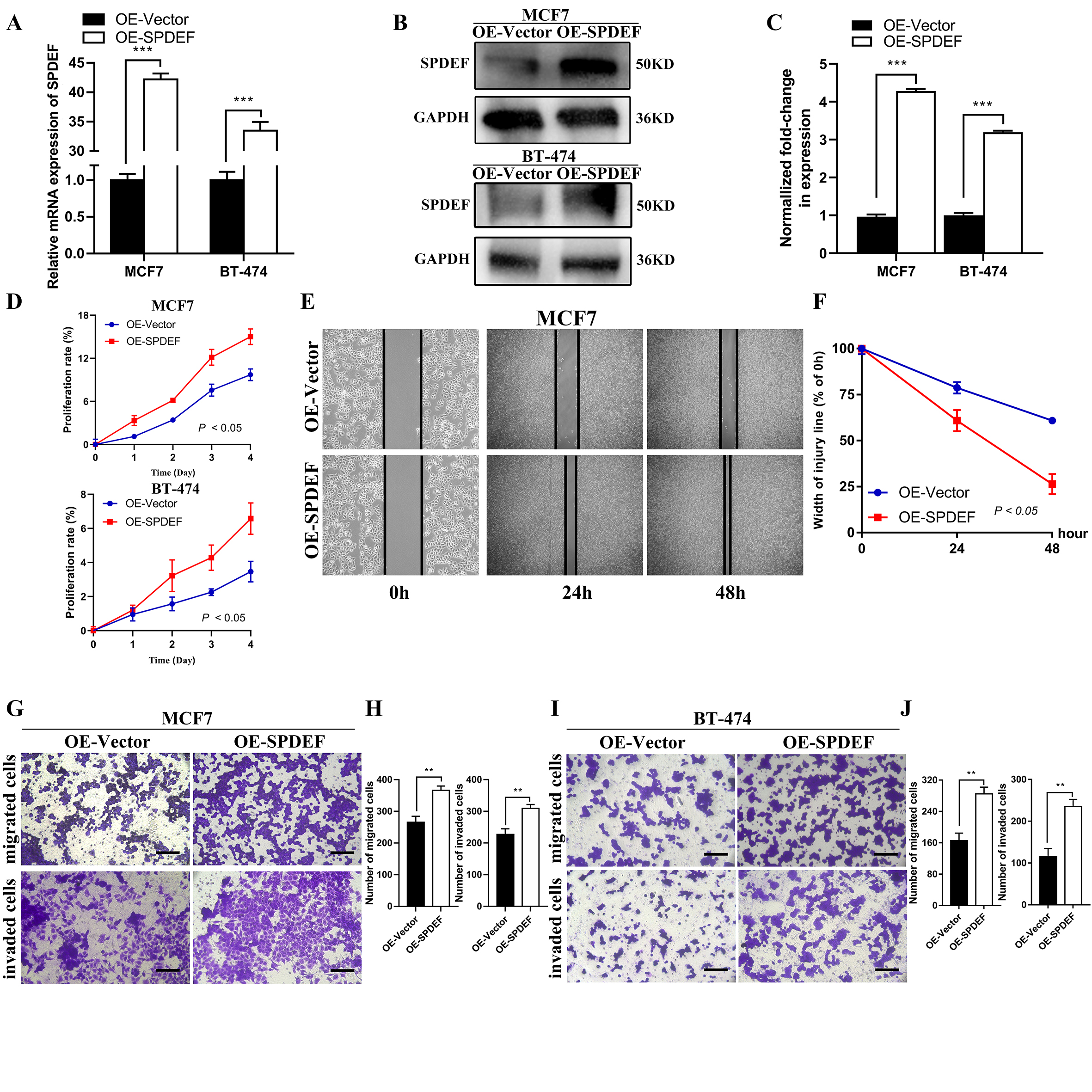

Supplement: Supplementary file 3 — supplement Figure S2 [file 41419_2023_6098_MOESM3_ESM.jpg]

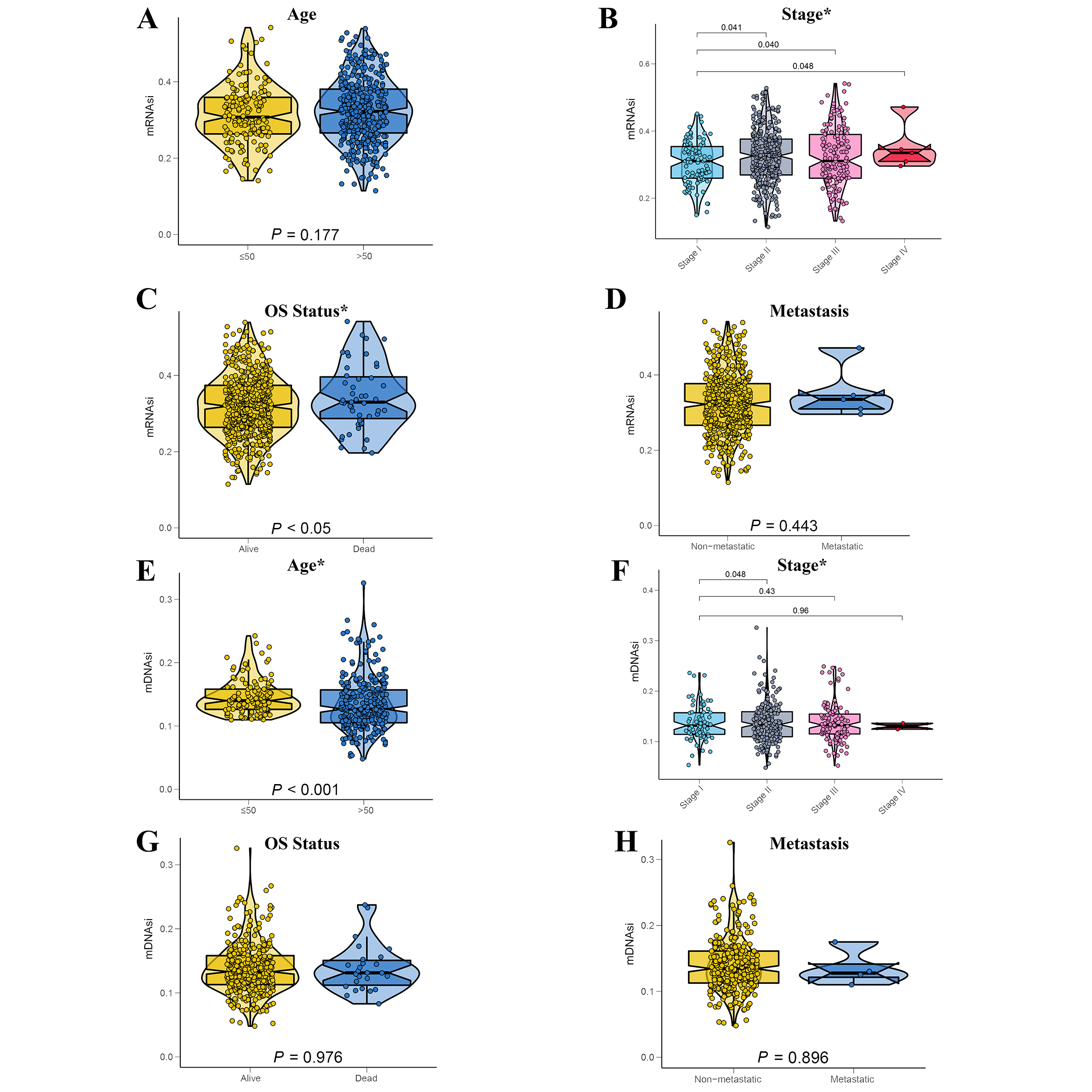

Supplement: Supplementary file 4 — supplement Figure S3 [file 41419_2023_6098_MOESM4_ESM.jpg]

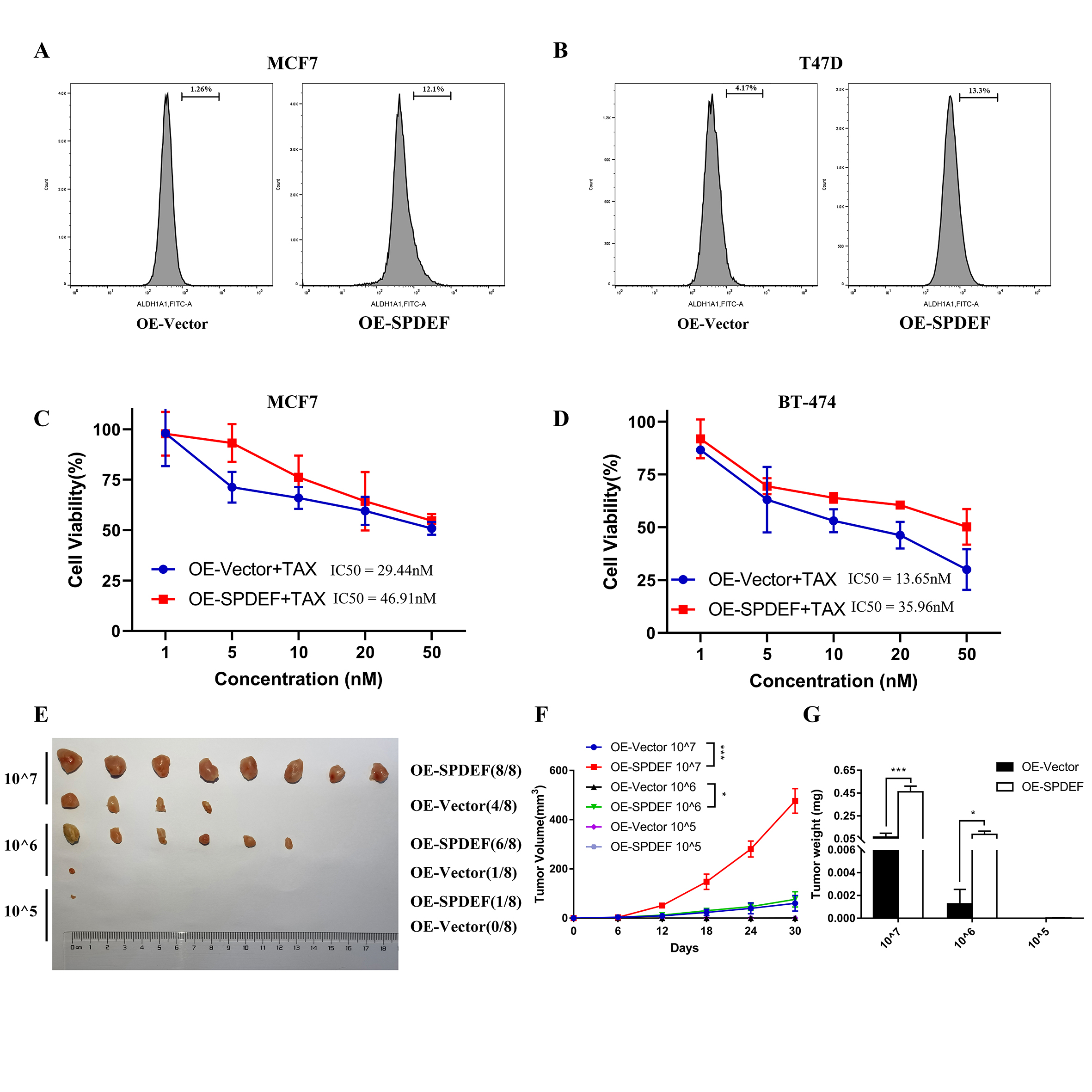

Supplement: Supplementary file 5 — supplement Figure S4 [file 41419_2023_6098_MOESM5_ESM.jpg]

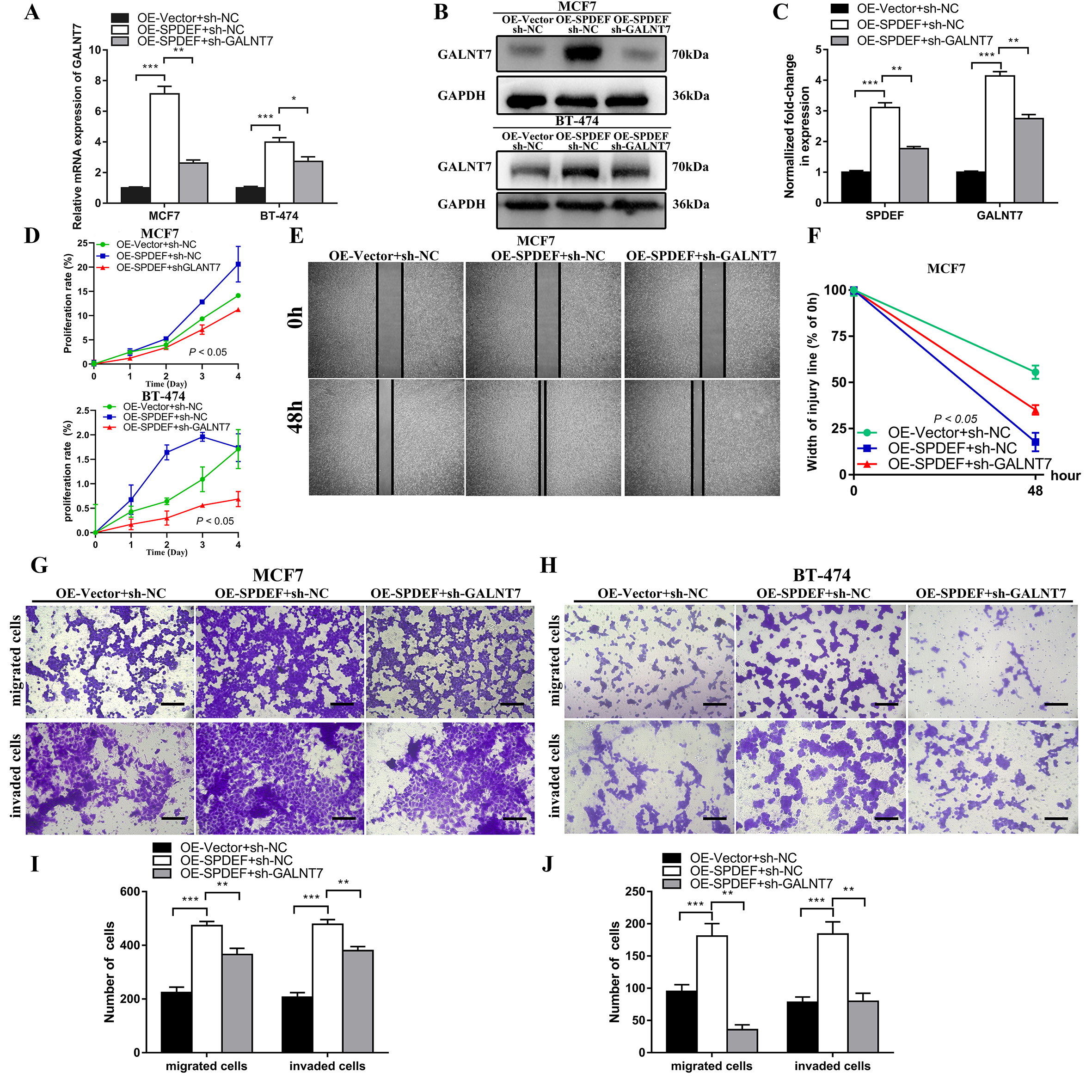

Supplement: Supplementary file 6 — supplement Figure S5 [file 41419_2023_6098_MOESM6_ESM.jpg]

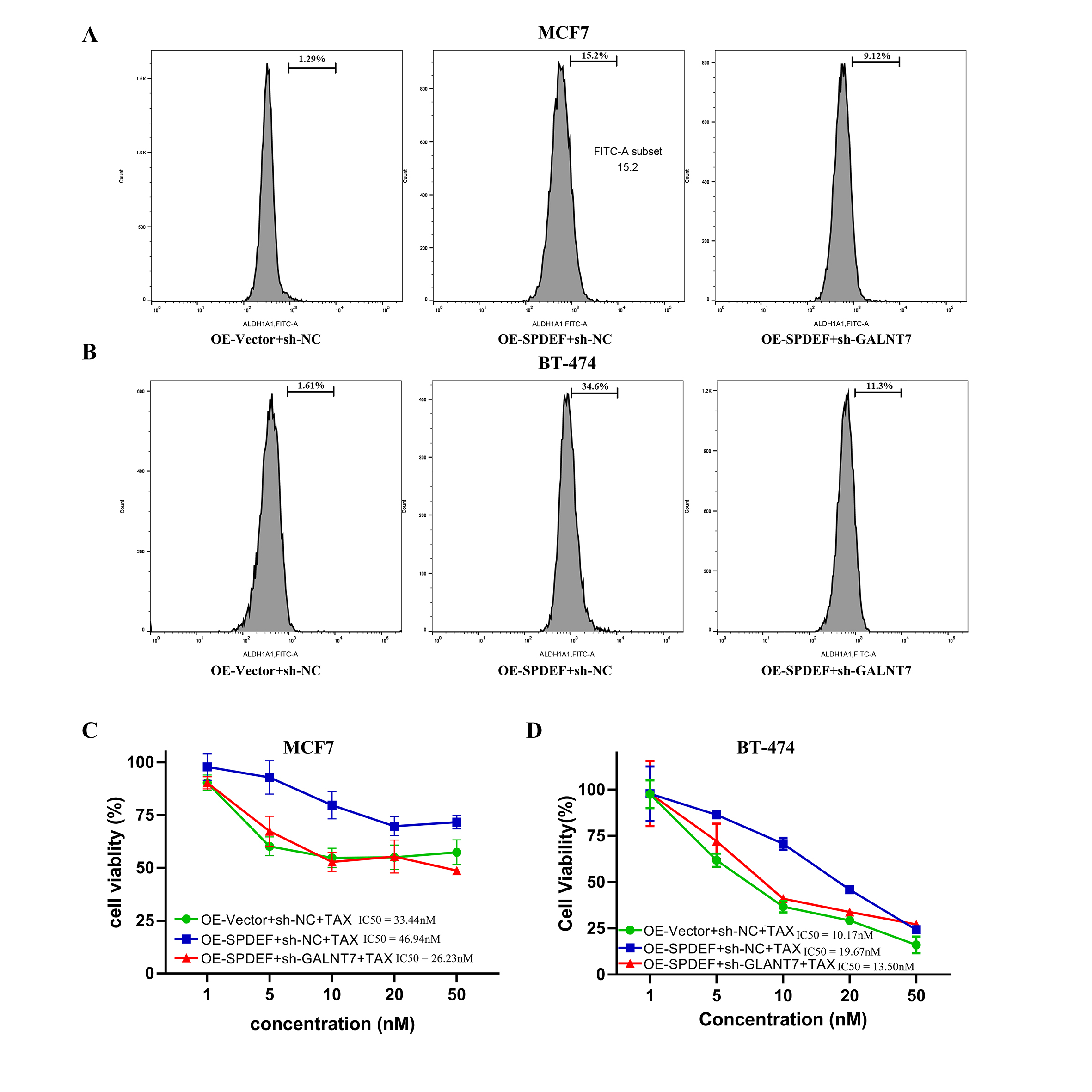

Supplement: Supplementary file 7 — supplement Figure S6 [file 41419_2023_6098_MOESM7_ESM.jpg]

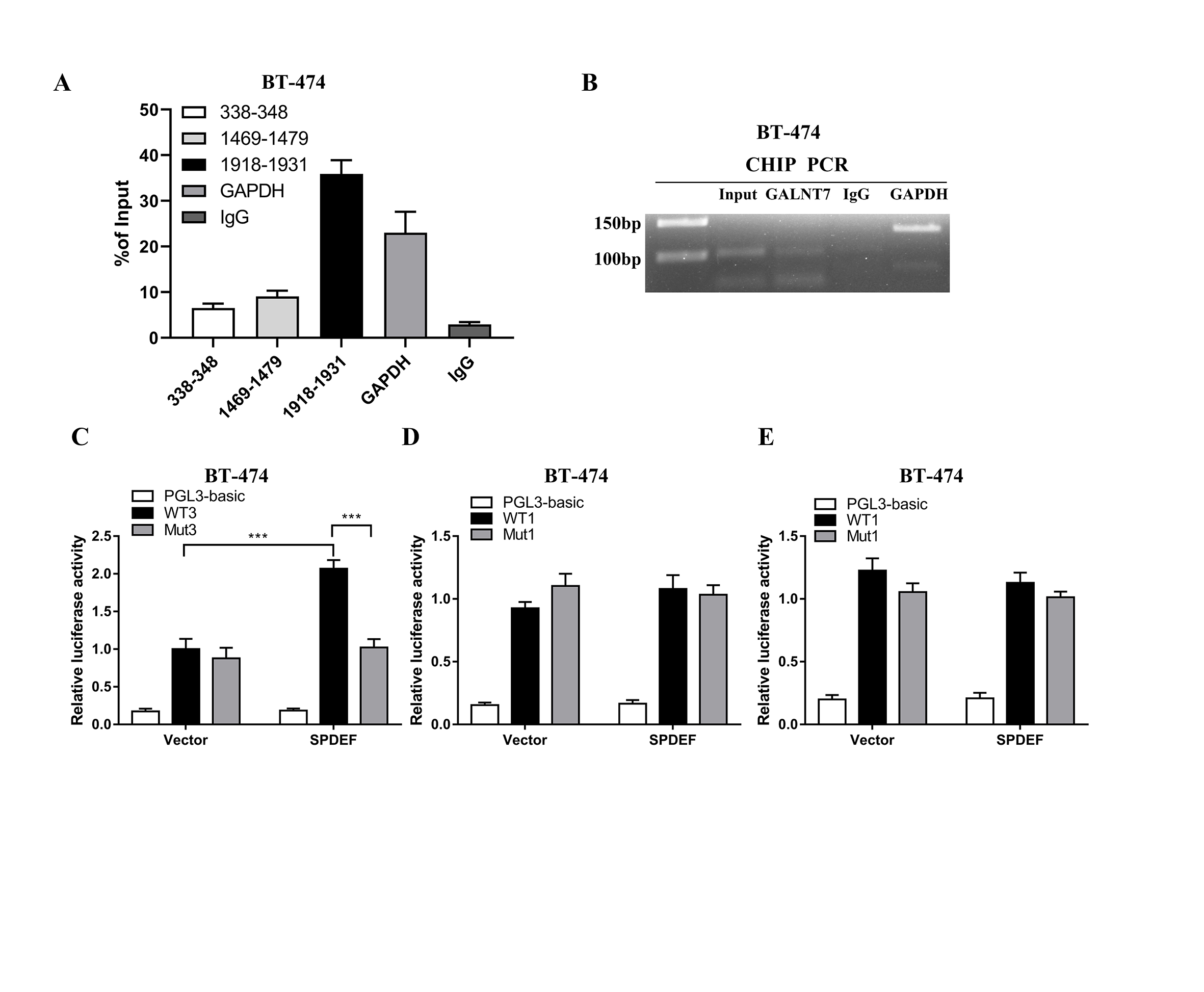

Supplement: Supplementary file 8 — supplement Figure S7 [file 41419_2023_6098_MOESM8_ESM.jpg]

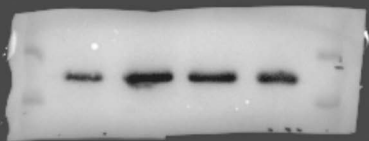

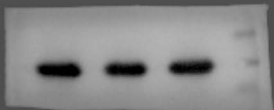

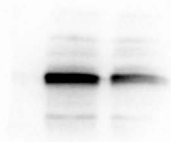

--

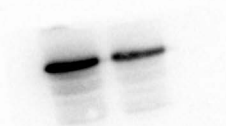

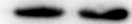

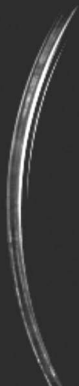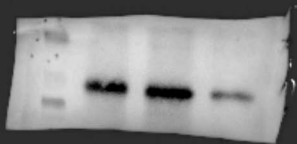

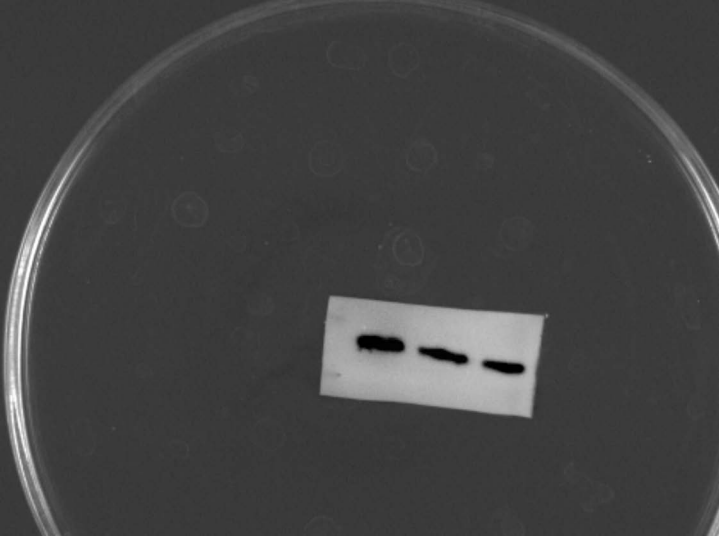

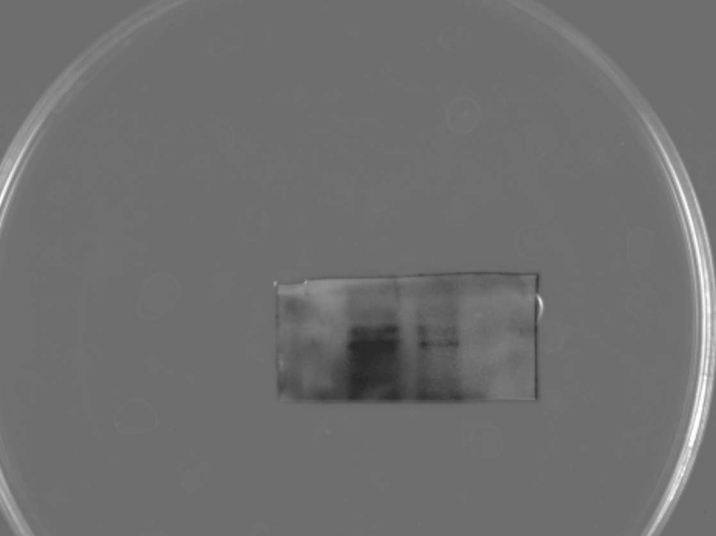

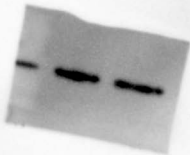

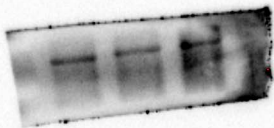

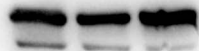

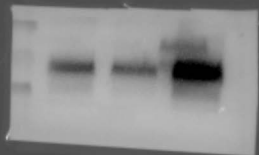

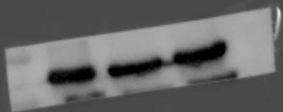

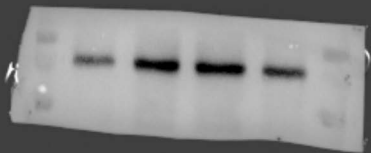

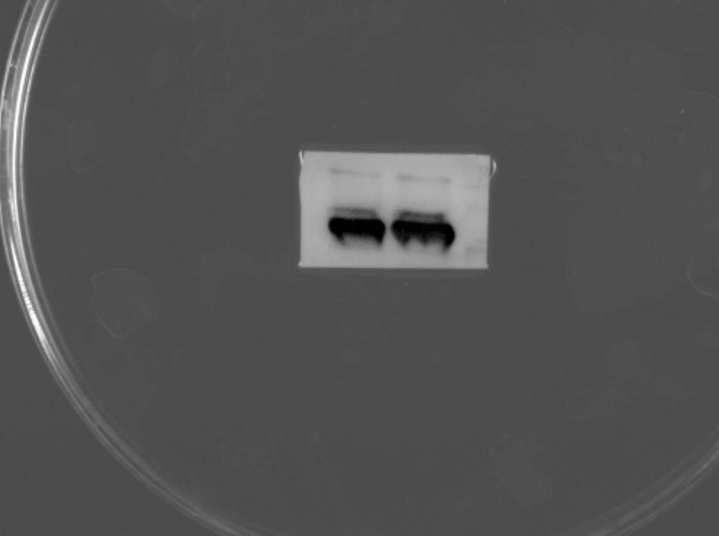

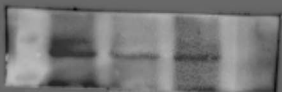

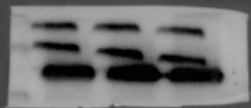

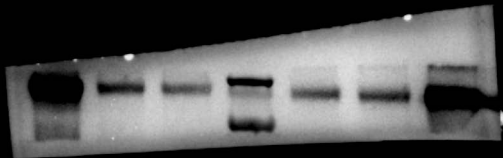

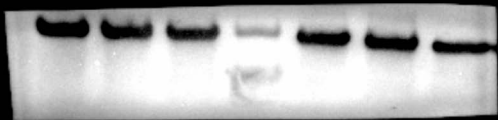

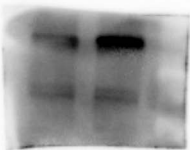

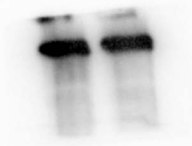

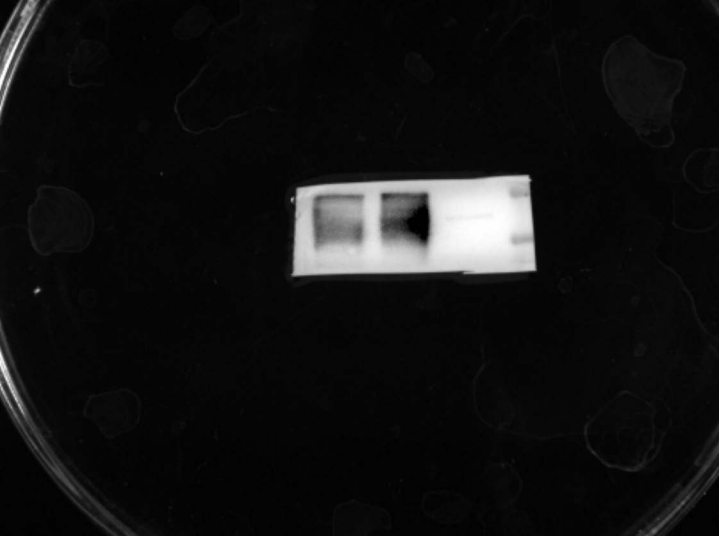

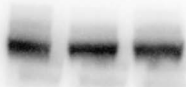

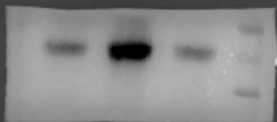

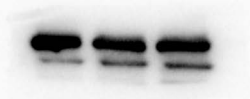

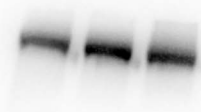

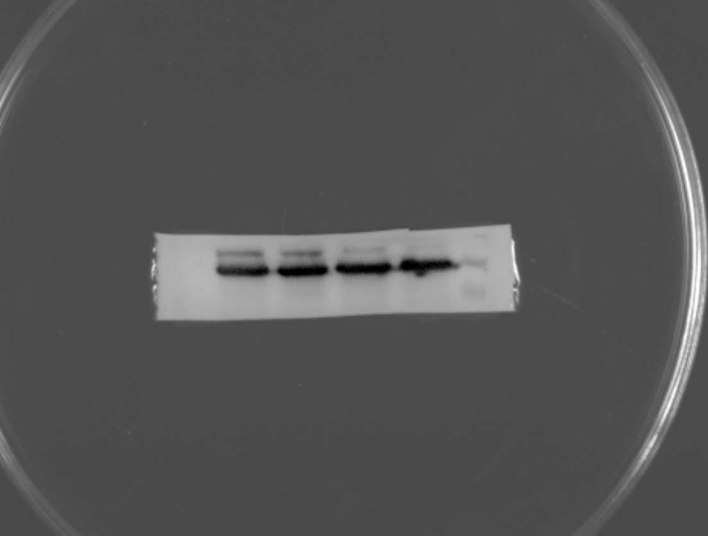

Supplement: Supplementary file 13 — western blot [file 41419_2023_6098_MOESM13_ESM.pdf]
