# Supplementary material for: SPDEF enhances cancer stem cell-like properties and tumorigenesis through directly promoting GALNT7 transcription in luminal breast cancer
Source: Cell Death Dis. 2023 Aug 26;14(8):569. doi: 10.1038/s41419-023-06098-z (PMC10460425; doi:10.1038/s41419-023-06098-z)
Supplement: Supplementary file 9 — supplement TableS1 [file 41419_2023_6098_MOESM9_ESM.docx]

**Table S1. Primers for RT‐qPCR**

| Gene name | Forward primers | Reverse primers |
| --- | --- | --- |
| SPDEF | GAGCCACCTGAGGAGCCTGAG | CTTGAGCACTTCGCCCACCAC |
| GALNT7 | GGCTAGTGGTCCTCTGGTCTTCC | CGGTTGGGCATGGGGTCATTG |
| NANOG | ACACTGGCTGAATCCTTCCTCTCC | CGCTGATTAGGCTCCAACCATACTC |
| MYC | AGCAGCGACTCTGAGGAGGAAC | TCCAGCAGAAGGTGATCCAGACTC |
| SOX2 | CAGCATGTCCTACTCGCAGCAG | CTGGAGTGGGAGGAAGAGGTAACC |
| OCT4 | GTGGTCCGAGTGTGGTTCTGTAAC | CCCAGCAGCCTCAAAATCCTCTC |
| BMI1 | GATGGCTGGTAAGATGGCGGTTC | GCACCTCCCAAGCGATGTTCAG |
| NES | AGAGCGAGCAGGAGGAGTTGG | GGAGTGGAGTCTGGAAGGGTCTC |
| CD44 | GGGAGTCAAGAAGGTGGAGCAAAC | GCCAAGAGGGATGCCAAGATGATC |
| TPB | CCGGAATCCCTATCTTTAGTCC | GCCTTTGTTGCTCTTCCAAAAT |
