# Supplementary material for: SPDEF enhances cancer stem cell-like properties and tumorigenesis through directly promoting GALNT7 transcription in luminal breast cancer
Source: Cell Death Dis. 2023 Aug 26;14(8):569. doi: 10.1038/s41419-023-06098-z (PMC10460425; doi:10.1038/s41419-023-06098-z)
Supplement: Supplementary file 10 — supplement TableS2 [file 41419_2023_6098_MOESM10_ESM.docx]

| Binding Sites | Forward primers | Reverse primers |
| --- | --- | --- |
| 338-348 | ACAGTCCTTCAGCCAACCAA | ACCAAACTACCTTCATGTGCTT |
| 1469-1479 | GCTATAAATCAGCCACGTGGAGC | TGCACCTCTCCCTTAGACCC |
| 1918-1931 | GGAGGAGCGGAGGGAAGAG | CTACGGCTGCCGCCCTCT |
| NC | CGTATTCCTCTAAGCCACAA | CGTATTCCTCTAAGCCACAA |

**Table S2. PCR Primer Sequence for ChIP-PCR**
