# Supplementary material for: SPDEF enhances cancer stem cell-like properties and tumorigenesis through directly promoting GALNT7 transcription in luminal breast cancer
Source: Cell Death Dis. 2023 Aug 26;14(8):569. doi: 10.1038/s41419-023-06098-z (PMC10460425; doi:10.1038/s41419-023-06098-z)
Supplement: Supplementary file 11 — supplement Table S3 [file 41419_2023_6098_MOESM11_ESM.docx]

**Table S3. Relationships between the protein expression of *SPDEF* and clinicopathological parameters in luminal BC based on IHC detection**

| **Variables** | ***SPDEF* mRNA expression of luminal BC** | | | *P*-value |
| --- | --- | --- | --- | --- |
|  | Total  (n=69) | Low (n=34) | High(n=35) |  |
| **Age at surgery** |  |  |  |  |
| <51 | 29 | 14 | 15 | 0.888 |
| ≥51 | 40 | 20 | 20 |  |
| **cTNM Stage** |  |  |  |  |
| Ⅰ + Ⅱ | 38 | 20 | 18 | 0.536 |
| Ⅲ + Ⅳ | 31 | 14 | 17 |  |
| **Lymphatic metastasis** |  |  |  |  |
| - | 27 | 15 | 12 | 0.403 |
| + | 42 | 19 | 23 |  |
| **Distant metastasis** |  |  |  |  |
| M0 | 63 | 32 | 31 | 0.414 |
| M1 | 6 | 2 | 4 |  |
