# Supplementary material for: SPDEF enhances cancer stem cell-like properties and tumorigenesis through directly promoting GALNT7 transcription in luminal breast cancer
Source: Cell Death Dis. 2023 Aug 26;14(8):569. doi: 10.1038/s41419-023-06098-z (PMC10460425; doi:10.1038/s41419-023-06098-z)
Supplement: Supplementary file 12 — supplement Table S4 [file 41419_2023_6098_MOESM12_ESM.docx]

**Table S4. Relationships between the expression of *GALNT7* and clinicopathological parameters in luminal BC based on ELISA**

| **Variables** | ***GALNT7* mRNA expression of luminal BC** | | | *P*-value |
| --- | --- | --- | --- | --- |
|  | Total  (n=31) | Low (n=16) | High (n=15) |  |
| **Age at surgery** |  |  |  |  |
| <51 | 11 | 6 | 5 | 0.809 |
| ≥51 | 20 | 10 | 10 |  |
| **cTNM Stage** |  |  |  |  |
| Ⅰ + Ⅱ | 17 | 11 | 6 | 0.108 |
| Ⅲ + Ⅳ | 14 | 5 | 9 |  |
| **Lymphatic metastasis** |  |  |  |  |
| - | 17 | 11 | 6 | 0.108 |
| + | 14 | 5 | 9 |  |
| **Distant metastasis** |  |  |  |  |
| M0 | 28 | 16 | 12 | 0.059 |
| M1 | 3 | 0 | 3 |  |
